# Robotic Radiosurgical Boost After Whole-Brain Radiotherapy for 12 Brain Metastases: En Bloc Consecutive Irradiation With Comprehensively Optimized Single Plan for Eight Lesions Totaling 118 cc

**DOI:** 10.7759/cureus.51367

**Published:** 2023-12-30

**Authors:** Kazuhiro Ohtakara, Kuniaki Tanahashi, Takehiro Yamada, Nobuyuki Tsunoda, Kojiro Suzuki

**Affiliations:** 1 Department of Radiation Oncology, Kainan Hospital Aichi Prefectural Welfare Federation of Agricultural Cooperatives, Yatomi, JPN; 2 Department of Radiology, Aichi Medical University, Nagakute, JPN; 3 Department of Neurosurgery, Gifu Prefectural Tajimi Hospital, Tajimi, JPN; 4 Department of Neurosurgery, Nagoya University Graduate School of Medicine, Nagoya, JPN; 5 Department of Radiology, Nagoya University Hospital, Nagoya, JPN; 6 Department of Breast Surgery, Japanese Red Cross Aichi Medical Center Nagoya Daiichi Hospital, Nagoya, JPN; 7 Department of Breast and Endocrine Surgery, Nagoya University Graduate School of Medicine, Nagoya, JPN

**Keywords:** biologically effective dose, brain metastases, large tumor, breast cancer, whole-brain radiotherapy, multiple tumors, robotic radiosurgery, stereotactic radiosurgery, fractionation, radiosurgical boost

## Abstract

General radiotherapeutic management for >10 brain metastases (BMs) totaling >100 cm^3^, including multiple large lesions (>10-30 cm^3^) in close proximity, demonstrated limited efficacy and/or safety. We describe a case of 12 BMs, summating 122.2 cm^3^, including a 39.6 cm^3^ maximum lesion and adjacent ones. The patient had an 8.1-year treatment history for recurrent/metastatic breast cancer refractory to endocrine and chemotherapy. BMs were treated with conventional whole-brain radiotherapy (WBRT) with 30 Gy/10 fractions (fr), followed by an immediate stereotactic radiosurgery (SRS) boost with 27 Gy/5 fr (52-64% isodoses) which covers the gross tumor boundaries of selected eight lesions (total 118.4 cm^3^). The SRS dose was defined to ensure the cumulative biologically effective dose (BED_10_) of just ≥80 Gy while minimizing the risk of radiation injury. The SRS was performed using a CyberKnife (CK) robotic system (Accuray Incorporated, Sunnyvale, California, United States) with a variable-sized collimator (10-40 mm), for which en bloc consecutive irradiation, using 215 beams based on a comprehensively optimized single plan (path), was adopted. The treatment time per fraction was ≤45 min (mean 5.6 min per lesion). Afterward, BMs demonstrated remarkable regression over six months, causing the total residual visible lesions of 12.6 cm^3^ (10.3%) at 11.4 months, despite the absence of obvious lesion shrinkage during the radiotherapy. WBRT, followed by an immediate 5-fr SRS boost with a total BED_10_ of 80 Gy to large and/or culprit lesions, can be an efficacious and safe treatment option for multiple BMs, totaling >120 cm^3^. En bloc consecutive irradiation with a single path provides overwhelmingly more efficient delivery for treating multiple lesions using CK in terms of irradiation time and comprehensive reduction of normal brain dose compared to individual planning. Volumetric-modulated arc-based >10-fr SRS with simultaneously integrated reduced-dose WBRT may be an alternative to further enhance efficacy and safety.

## Introduction

Brain metastases (BMs) are a major cause of morbidity and mortality from metastatic breast cancer (MBC) [1]. Human epidermal growth factor receptor-2 (HER2)-overexpressing and triple-negative MBC demonstrated a higher incidence of BM, compared to hormone receptor-positive (HR+) and HER2-non-overexpressing (HER2-) subtypes [2,3]. However, screening of presymptomatic BMs in patients with breast cancer (BC) is uncommon, at either the initial staging or re-staging following recurrence, compared to lung cancer or malignant melanoma with a high predisposition to developing BMs [1-3]. BMs gradually grow to a significant volume without remarkable parenchymal edema, while their proliferation is suppressed to some extent by systemic therapy that penetrates the blood-tumor barriers [4,5]. Thus, MBC-induced BMs are frequently diagnosed only in a substantially advanced stage, such as large volumes, in large numbers, and/or dissemination when the related symptoms become obvious [5].

Conventional external beam radiotherapy (EBRT), including whole-brain radiotherapy (WBRT) and stereotactic radiosurgery (SRS), indicates limited efficacy and/or safety for multiple BMs totaling >100 cm^3^, including several >10-30 cm^3^ lesions and multiple in close proximity [6,7]. In particular, such BMs, which are associated with multiple significant mass effects and the risk of spontaneous or iatrogenic imminent herniation, pose a challenge.

The CyberKnife^®^ (CK) system (Accuray Incorporated, Sunnyvale, California, United States) is a robotic platform that is dedicated to high-precision frameless SRS for intracranial and other versatile locations [8]. Meanwhile, the treatment planning for multiple BMs is usually performed for each lesion, and the assigned dose adjustments (re-scaling) are required for each lesion, considering the dose interference from irradiation on the same day. The more lesions are irradiated on the same day, the more complicated the treatment plan becomes. The treatment time per fraction for ≥4 lesions frequently requires ≥60-80 min, depending on the dose per fraction, due to a super multiple static-beam delivery with fixed beam apertures for each plan (path). These limitations had somewhat compromised an assertive adaptation of CK for >4 BMs.

Herein, we describe a case of systemic therapy-refractory HR+/HER2- MBC-derived BMs with 12 lesions totaling 122.2 cm^3^, including a 39.6 cm^3^ maximum lesion and adjacent ones. BMs were treated with conventional WBRT (30 Gy/10 fr) followed by an immediate 5-fr SRS boost using CK to the selected eight lesions, summating 118.4 cm^3^, to sufficiently and immediately alleviate the relevant symptoms while minimizing adverse radiation effects (AREs). The SRS dose was defined to ensure that the cumulative biologically effective dose (BED) of the gross tumor boundaries was ≥80 Gy [8,9]. En bloc consecutive irradiation, based on a comprehensively optimized single plan, was adopted using a variable-sized collimator, instead of an individual planning, to efficiently irradiate the eight lesions using CK. We demonstrate the efficacy and safety of the radiotherapeutic scheme, with the significance of the delivery technique for irradiating multiple lesions using CK. We discuss how to further enhance the efficacy and safety of the radiotherapeutic management.

Part of this report was previously presented at the 30th Annual Meeting of the Japanese Society of Stereotactic Radiosurgery, held online on June 12, 2021.

## Case presentation

**Figure 1:**
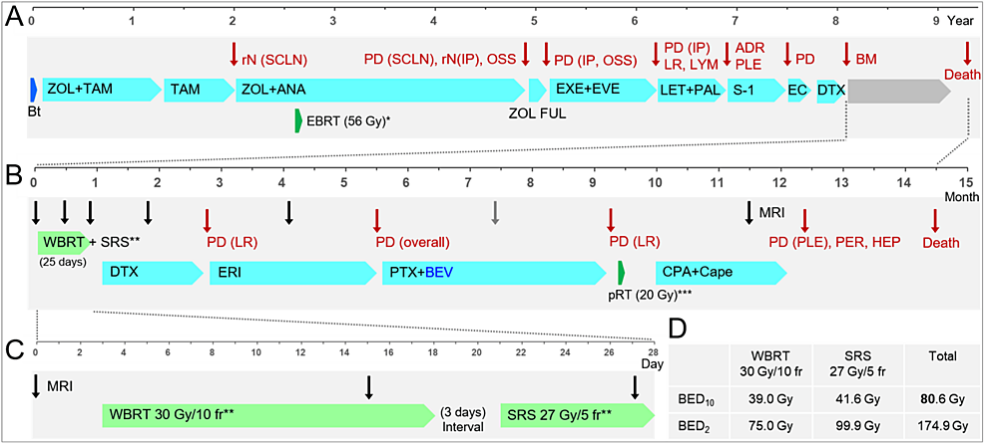
Summary of anti-cancer treatments, patterns of failures, and the acquisition timings of brain magnetic resonance images. The figure shows (A) the treatment progress (in years) mainly until the diagnosis of BMs, (B) the treatment progress (in months) after the BM diagnosis, (C) the schedule (in days) for EBRT for BMs, and (D) the biologically effective doses of the EBRT. *EBRT: conventional 3D-CRT in 56 Gy/28 fr for enhancing the locoregional disease control including the supraclavicular lymph node metastases. **The WBRT and SRS were performed over 15 days and seven days, including five days off and two days off, respectively. ***pRT: palliative 3D-CRT with 20 Gy/5 fr for the chest wall recurrence refractory to chemotherapy. BMs: brain metastases; EBRT: external beam radiotherapy; 3D-CRT: three-dimensional conformal radiotherapy; rN: regional lymph node recurrence; SCLN: supraclavicular lymph nodes; PD: progression; IP: interpectoral (Rotter) LN; OSS: bone metastasis; LR: local recurrence; LYM: distant lymph node metastases; ADR: adrenal metastasis; PLE: pleural metastases/dissemination; Bt: mastectomy; ZOL: goserelin; TAM: tamoxifen; ANA: anastrozole; FUL: fulvestrant; EXE: exemestane; EVE: everolimus; LET: letrozole; PAL: palbociclib; EC: epirubicin+cyclophosphamide (CPA); DTX: docetaxel; MRI: magnetic resonance imaging; WBRT: whole-brain radiotherapy; SRS: stereotactic radiosurgery; PER: peritoneal dissemination; HEP: liver metastases; ERI: eribulin; PTX: paclitaxel; BEV: bevacizumab; pRT: palliative radiotherapy; Cape: capecitabine; fr: fractions; BED_10/2_ : a biologically effective dose based on the linear-quadratic model with an alpha/beta ratio of 10/2

A 57-year-old female patient with an 8.1-year treatment history of BC was diagnosed with multiple brain lesions after a vehicular accident. The patient remembered nothing regarding the accident. The patient had recently become slower and less communicative, and she frequently bumped into objects in the days before the accident. The Karnofsky performance status (KPS) was 60. The previous treatments for BC are summarized in Figure 1A.

**Figure 2:**
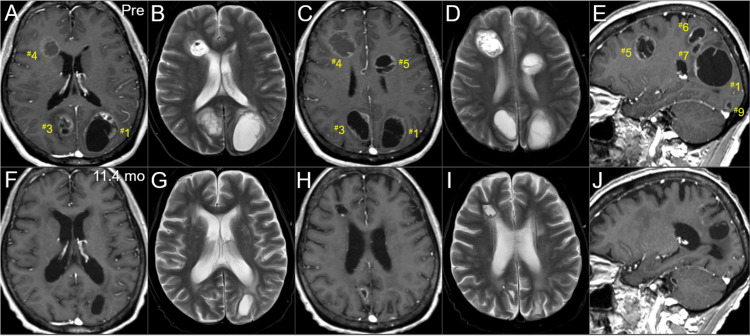
Magnetic resonance images at the diagnosis and last examination, mainly focusing on the boost lesions in the left hemisphere. The images show CE T1-WIs (A, C, E, F, H, J); T2-WIs (B, D, G, I); axial images (A-D, F-I); sagittal images in the left hemispheres (E, J); three days before the initiation of WBRT (pre) (A-E); and 11.4 months after the WBRT initiation (F-J). (A-J) These images are shown at the same magnification and coordinates under co-registration and fusions based on the pre-irradiation images (A, B, F, G: almost the same cross-section; C, D, G, H: the same section, E, J: the same section). Therefore, some images with thick slices are slightly blurry (D, G, I). (A-E) The numbers with ^#^ assigned to each lesion correspond to the lesion numbers in Table 1. Seven lesions are observed, most of them cystic, with some solid components (^#^1,3,4,5). The perilesional edemas before irradiation are minimal considering the lesion volumes. (E) Three lesions (^#^1,6,7) in the left parietal lobe are contiguous and adjacent. (F-J) At 11.4 months, all the seven lesions had remarkably regressed or almost disappeared. CE: contrast-enhanced; WIs: weighted images; WBRT: whole-brain radiotherapy

**Figure 3:**
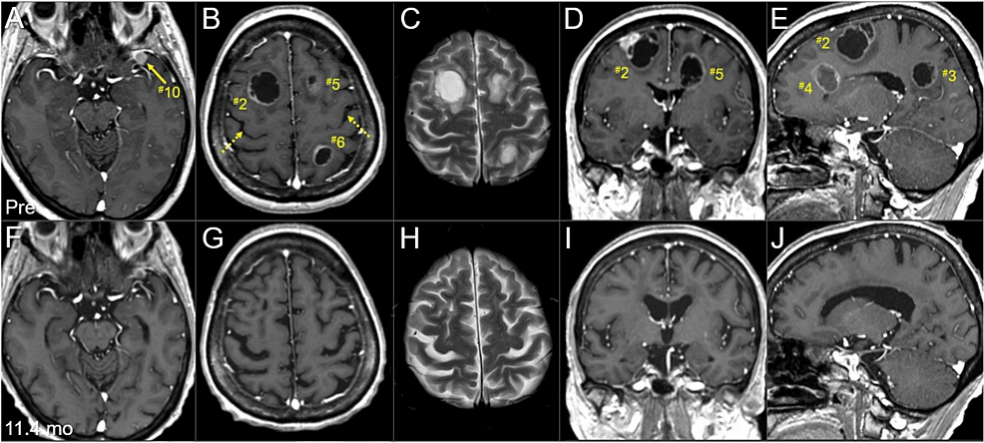
Magnetic resonance images at the diagnosis and last examination, mainly focusing on the boost lesions in the right hemisphere. The images show CE T1-WIs (A, C, E, G, I, K); T2-WIs (B, D, H, J); axial images (A-C, F-H); coronal images (D, I); sagittal images in the right hemispheres (E, J); three days before the initiation of WBRT (pre) (A-E); and 11.4 months after the WBRT initiation (F-J). (A-J) These images are shown at the same magnification and coordinates under co-registration and fusions based on the pre-irradiation images (A, F: the same cross-section; B, C, G, H: the same section). (A-E) Six lesions are observed, most of them cystic, with some solid components (^#^2,4,5). An extra-parenchymal dura-based lesion (#10) is solid. (B) The central sulci are indicated by dashed arrows. (E) Two large lesions (^#^2,4) in the right frontal lobe are in close proximity. (F-J) At 11.4 months, all the seven lesions had remarkably regressed or almost disappeared. CE: contrast-enhanced; WIs: weighted images; WBRT: whole-brain radiotherapy

**Figure 4:**
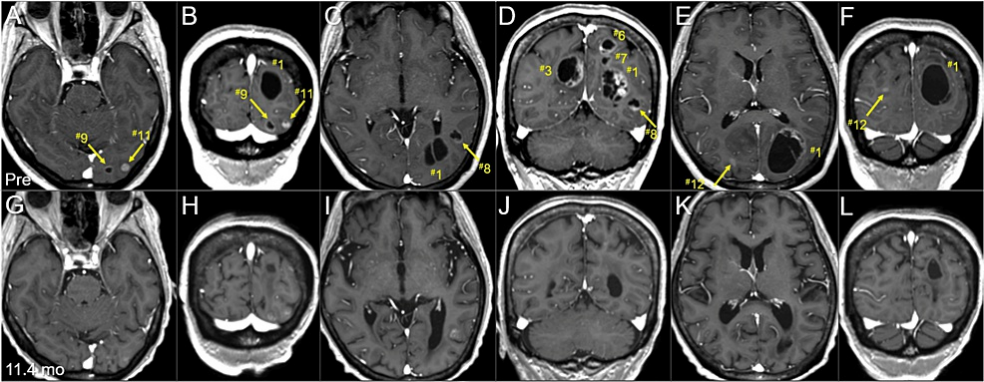
Magnetic resonance images at the diagnosis and last examination, focusing on the four non-boost lesions. The images show CE T1-WIs (A-L); axial images (A, C, E, G, I, K); coronal images (B, D, F, H, J, L); three days before the initiation of WBRT (pre) (A-F); and 11.4 months after the WBRT initiation (G-L). (A-L) These images are shown at the same magnification and coordinates under co-registration and fusions based on the pre-irradiation images. (A-F) Eight lesions are observed, most of them cystic, with some solid components (^#^1,3,6). Two small lesions (^#^11,12) are mainly solid. (G-L) At 11.4 months, all the four non-boost lesions (^#^8,9,11,12) had regressed or almost disappeared. CE: contrast-enhanced; WIs: weighted images; WBRT: whole-brain radiotherapy

The BC was a HR+ and HER2- subtype and harbored no breast cancer gene (BRCA) 1/2 mutations. The patient had received the third-line chemotherapy for MBC with visceral involvement, refractory to endocrine therapy, before the accident. Extracranial active disease was generally controlled. Conventional magnetic resonance images (MRI) revealed 12 enhancing lesions that were mainly cystic and associated with minimal perilesional edemas, considering the volumes (Figure 2, Figure 3, Figure 4, and Table 1).

**Table 1:** Planning parameters for the radiosurgical boost and lesion volume changes. Lesions are numbered in descending order of GTV. *Cystic lesions without enhancement or scar-like enhancing lesions that are deemed to be in complete remission are contoured as GTVs. **The lesions ^#^6 and 7 were adjacent and their planning parameters are shown as one lesion. ***The eight lesions receiving SRS boost have a total of 118.41 cm^3^. No: number; GTV: gross tumor volume; pre: three days before the whole-brain radiotherapy; D_min_: minimum dose; BED_10_: a biologically effective dose based on the linear-quadratic formula with an alpha/beta ratio of 10; %IDS: isodose surface (%) relative to the maximum dose (100%); D_max_: maximum dose; Rt.: right; Lt.: left

No	Location	Boost	GTV (pre)	D_min_ (BED_10_)	27 Gy coverage	%IDS to D_max_	GTV* (last)
^#^1	Lt. parietal	+	39.56 cm^3^	22 Gy	95.3%	52.8%	4.75 cm^3^ (12%)
^#^2	Rt. frontal	+	21.61 cm^3^	22.3 Gy	95.6%	51.9%	1.40 cm^3^ (6.5%)
^#^3	Rt. parietal	+	19.49 cm^3^	24.1 Gy	97.6%	54.1%	1.62 cm^3^ (8.3%)
^#^4	Rt. frontal	+	15.34 cm^3^	22.6 Gy	96.5%	55.8%	2 cm^3^ (13%)
^#^5	Lt. frontal	+	14.83 cm^3^	23.3 Gy	96.7%	55%	1.32 cm^3^ (8.9%)
^#^6	Lt. parietal	+	4.75 cm^3^	24 Gy**	98.2%**	54%**	0.11 cm^3^ (1.6%)
^#^7	Lt. parietal	+	2.02 cm^3^	(24 Gy)**	(98.2%)**	(54%)**	0 cm^3^ (0%)
^#^8	Lt. temporal	-	1.99 cm^3^	-	-	-	0.40 cm^3^ (20.1%)
^#^9	Lt. occipital	-	1.07 cm^3^	-	-	-	0.48 cm^3^ (44.9%)
^#^10	Lt. temporal	+	0.81 cm^3^	26.1 Gy	99.3%	63.7%	0.38 cm^3^ (46.9%)
^#^11	Lt. occipital	-	0.46 cm^3^	-	-	-	0.13 cm^3^ (28.3%)
^#^12	Rt. parietal	-	0.22 cm^3^	-	-	-	0 cm^3^ (0%)
Total***	-	-	122.15 cm^3^	-	-	-	12.59 cm^3^ (10.3%)

**Figure 5:**
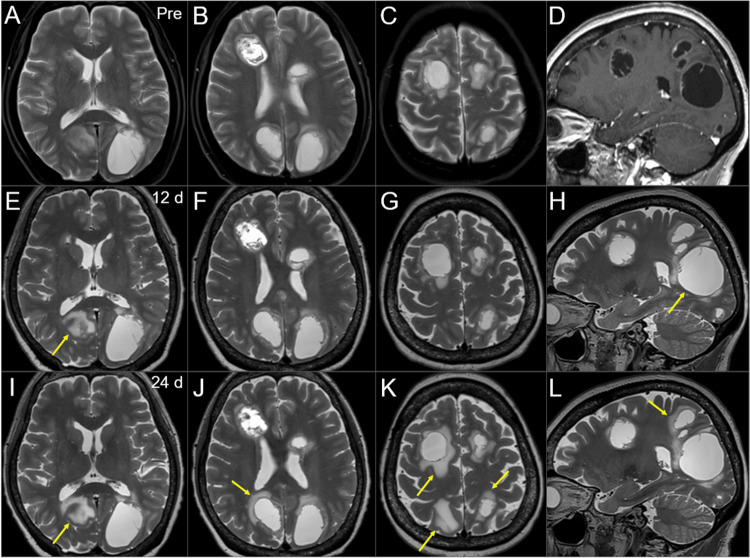
Magnetic resonance images during the 15-fr radiosurgery and subsequent WBRT. The images show T2-WIs (A-C, E-L); a CE T1-WI (sagittal T2-WIs before WBRT were unavailable) (D); axial images (A-C, E-G, I-K); sagittal images (D, H, L); three days before the initiation of WBRT (pre) (A-D); 12 days (d) after the WBRT initiation (at the 8 fr of WBRT) (E-H); and 24 days after the WBRT initiation (at the completion of SRS boost) (I-L). (A-H) These images are shown at the same magnification and coordinates under co-registration and fusion based on the pre-SRS images. (E-H) There was no obvious change in the tumor configuration of most lesions. The largest lesion was slightly enlarged (arrow in H). The perilesional edema aggravated in some lesions (arrow in E). (I-L) There is still no obvious change in the tumor configuration of almost all the lesions. The expansion of the largest lesion has not progressed. The perilesional edemas appear or aggravate in multiple lesions (arrows in I-L). WIs: weighted images; CE: contrast-enhanced; WBRT: whole-brain radiotherapy; SRS: stereotactic radiosurgery; fr: fraction

All the lesions were supratentorial, with the five lesions being ≥14.8 cm^3^. The maximum lesion in the left parietal lobe was 39.6 cm^3^ (48 mm in the maximum diameter) concomitant with two adjacent lesions (4.8 cm^3^, 2 cm^3^). The total volume of the 12 lesions was 122.2 cm^3^ (8.1 times of 15 cm^3^: the safe upper limit for single-fr SRS of multiple BMs) [7]. All the lesions developed and progressed while sparing the eloquent cortexes or deep critical structures including the brainstem and the pyramidal tracts. The development of >12 BMs refractory to endocrine and chemotherapy and the presence of a lesion in contact with the cerebrospinal fluid (CSF) space indicated a high potential for multiple subclinical lesions and CSF dissemination. Meanwhile, ≥10 fr SRS alone for multiple lesions, including large ones, in close proximity indicated a high risk of AREs [10]. Therefore, WBRT (30 Gy/10 fr) was initiated three days following the diagnosis (six days after the traffic accident) (Figure 1B and Figure 1C). A sequential SRS boost was scheduled immediately following the WBRT, for which MRI (1 mm slice thickness) was acquired at 8 fr of WBRT (12 days after the WBRT initiation), to sufficiently and promptly alleviate the relevant symptoms (Figure 5). 

Eight lesions that were large, culprit (causing symptoms), or in contact with the CSF space were selected for SRS boost, considering the absence of shrinking lesions during the WBRT (Table 1). The SRS boost was performed using a CK M6 treatment delivery system, where a linear accelerator is mounted on an industrial robot with a six-axis manipulator arm, producing 6 MV flattening-filter-free (FFF) X-rays at a dose rate of 1000 MU/min [8]. The gross tumor volumes (GTVs) were mainly defined on the enhancing lesions under T1/T2 matching [5]. The SRS dose was defined to ensure just over 80-Gy cumulative BED covering the GTV boundaries, in which the BED was based on the linear-quadratic formula at an alpha/beta ratio of 10 (BED_10_) [10]. The basic planning scheme was to cover GTVs with ≥27 Gy/5 fr (BED_10_ 41.6 Gy), where the dose gradients outside the GTVs were optimized as steep as possible, by assertively allowing the extremely high doses inside the GTVs [11,12]. The cumulative BED_10_ of the WBRT (30 Gy/10 fr) and SRS boost (27 Gy/5 fr) was 80.6 Gy (Figure 1D). We aimed to encompass ≥98% of the GTVs with 27 Gy, while the ≥95% GTV coverage was allowed for some large lesions (Table 1).

**Figure 6:**
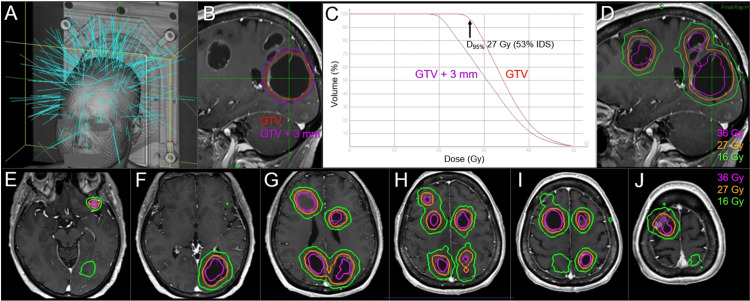
Room's eye view, target definitions, dose-volume histograms, and dose distributions for the radiosurgical boost. The images show a room's eye view (A); target definitions for the largest lesion (#1) (B); dose-volume histograms of the largest lesion (C); and representative dose distributions superimposed onto CE T1-WIs (D-J); sagittal views (B, D); and axial views (E-J). (A) The light blue lines indicate the directions of 215 beams. (C) 27 Gy corresponds to 53% IDS relative to the maximum dose (100%). (D-J) The cumulative BED_10_s (BED_2_s) of WBRT and SRS doses of 36 Gy and 16 Gy are 100.9 Gy (240.6 Gy) and 60.1 Gy (116.6 Gy), respectively. GTV: gross tumor volume; GTV+3 mm: GTV plus an isotropic 3-mm margin; D_95%_: a minimum dose covering at least 95% of the target volume; IDS: isodose surface; CE: contrast-enhanced; WIs: weighted images; BED_10/2_: a biologically effective dose based on the linear-quadratic formula with an alpha/beta ratio of 10/2

To efficiently irradiate the eight lesions using CK in a short time while comprehensively and efficiently minimizing the surrounding brain doses, en bloc consecutive irradiation based on a comprehensively optimized single plan (path) was adopted utilizing the Iris™ variable aperture collimator (Accuray Incorporated, Sunnyvale, California, United States), which composes of two hexagonal banks of tungsten segments that produce dodecagonal apertures (virtually circular) ranging from 5 mm to 60 mm in diameter [8]. In this plan, eight different diameters (10, 12.5, 15, 20, 25, 30, 35, 40 mm) were used, and multiple sizes were individually combined depending on the size and shape of the lesion. The optimization algorithm was the CK-VOLO^®^ (Accuray Incorporated, Sunnyvale, California, United States), built into the dedicated planning system Precision^®^ (Accuray Incorporated, Sunnyvale, California, United States), with a finite-sized pencil-beam model for dose calculation (1-mm grid size) [8]. The estimated treatment time (EST) was ≤45 minutes per fraction (mean 5.6 minutes per lesion), with 215 beams (mean 26.9 beams per lesion) from 98 nodes. The representative dose distributions, dose-volume histograms (DVHs) of the largest lesion, and planning parameters are shown in Figure 6 and Table 1.

The SRS boost was initiated three days after completing WBRT. MRI after SRS boost demonstrated no obvious tumor shrinkage, while perilesional edema development or aggravation was observed around some lesions (Figure 5) [8,13,14]. The patient's general condition was stable during the EBRT, probably due to the immediately administered steroids after the BM diagnosis.

**Figure 7:**
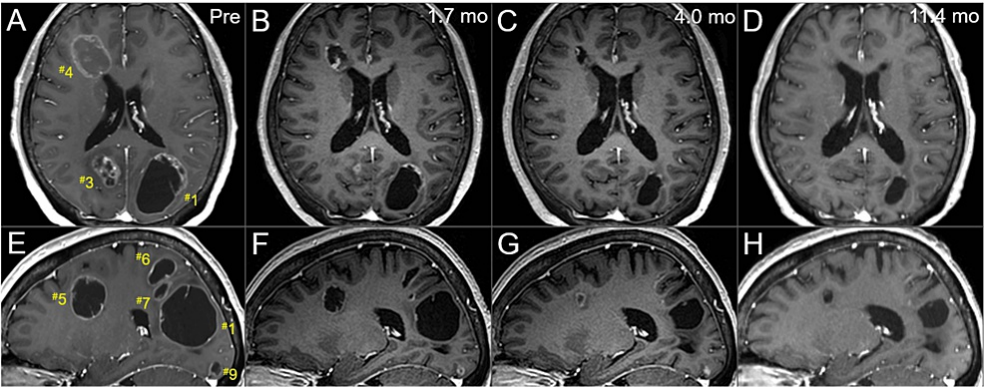
Serial magnetic resonance images before and after the radiotherapy, mainly focusing on the left hemisphere lesions. The images show CE-T1-WIs (A-H); axial images (A-D); sagittal images in the left hemispheres (E-H); before the SRS boost (at 8 fr of the WBRT) (A, E); at 1.7 months after the initiation of WBRT (29 days after the completion of SRS boost) (B, F); at 4 months after the WBRT initiation (C, G); and at 11.4 months (D, H). (A-H) These images are shown at the same magnification and coordinates under co-registration and fusion based on the pre-SRS images. All the seven lesions remarkably shrunk at 1.7 months and further regressed after that at 11.4 months, with the solid components almost disappearing. CE: contrast-enhanced; WIs: weighted images; SRS: stereotactic radiosurgery; fr: fractions; WBRT: whole-brain radiotherapy

**Figure 8:**
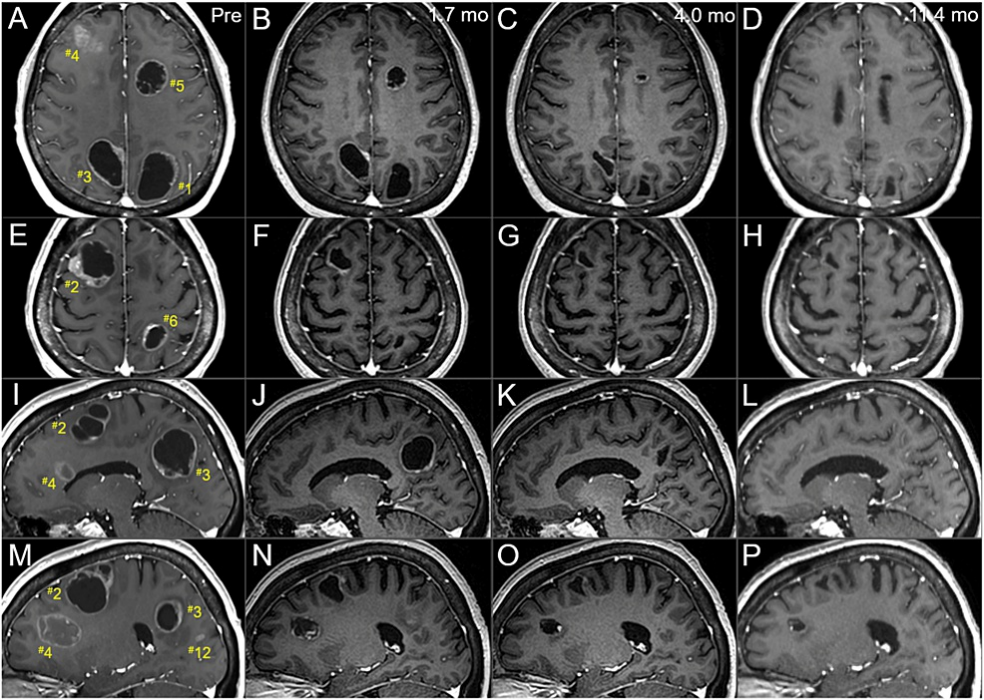
Serial magnetic resonance images before and after the radiotherapy, mainly focusing on the right hemisphere lesions. The images show CE T1-WIs (A-P); axial images (A-H); sagittal images in the right hemispheres (I-P); before the SRS boost (at 8 fr of the WBRT) (A, E, I, M); at 1.7 months after the initiation of WBRT (29 days after the completion of SRS boost) (B, F, J, N); at 4 months after the WBRT initiation (C, G, K, O); and at 11.4 months (D, H, L, P). (A-P) These images are shown at the same magnification and coordinates under co-registration and fusion based on the pre-SRS images. All the seven lesions remarkably shrunk at 1.7 months and further regressed after that at 11.4 months, with the solid components almost disappearing. CE: contrast-enhanced; WIs: weighted images; SRS: stereotactic radiosurgery; fr: fractions; WBRT: whole-brain radiotherapy

After completing the SRS boost, the patient gradually became more active and verbally communicated well (KPS ≥80); thus, chemotherapy was reinitiated (Figure 1B). MRI 29 days after the SRS completion (1.7 months after the WBRT initiation) revealed obvious tumor shrinkage overall (Figure 7 and Figure 8).

The patient developed left hearing impairment due to WBRT-induced otitis media 2.2 months after the WBRT completion. The BMs continued to gradually regress, and the relevant mass effects were remarkably attenuated for more than six months 1.7 months after WBRT initiation, causing total residual visible (mostly scar-like) lesions of 12.6 cm^3^ (10.3% of the initial total GTVs) at 11.4 months after WBRT initiation (Figure 7, Figure 8, and Table 1). Further, the four small non-boost lesions sustained regression (Figure 4), probably due to the additional efficacies of low-dose spillages from the SRS boost and sequential chemotherapies (Figure 1B).

Chemotherapy, after the cranial EBRT, was changed three times due to progressions of locoregional recurrences and metastatic lesions (Figure 1A). A chest wall recurrence was administered palliative EBRT (Figure 1B). However, the patient's general condition deteriorated after 12.3 months from the WBRT initiation due to hydronephrosis from tumoral ureteral obstruction and exacerbation of pleural dissemination concomitant with severe infectious pneumonia, causing systemic therapy discontinuation. The patient expired 14.5 months after the BM diagnosis (9.3 years after the initial breast surgery, 7.3 years after regional lymph node recurrence).

**Figure 9:**
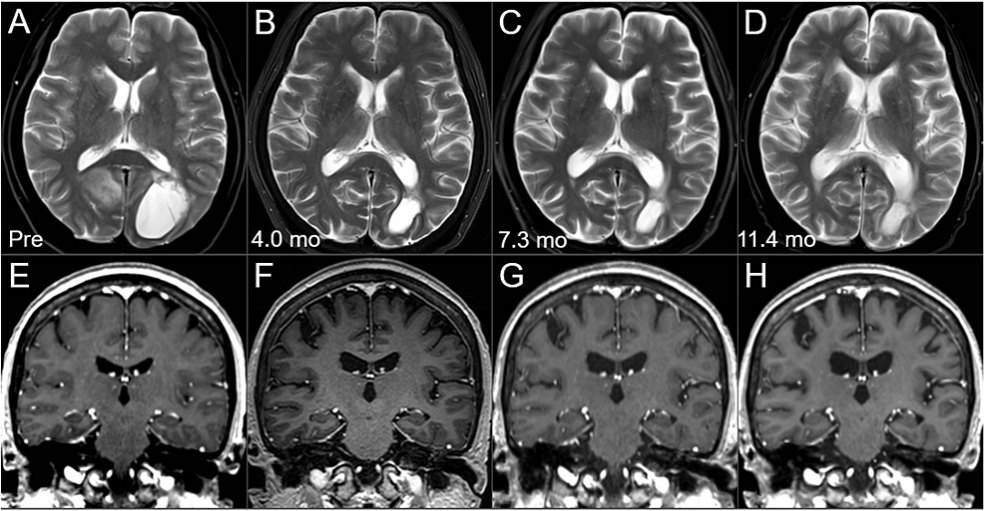
Morphological changes of the brain on magnetic resonance images before and after the external beam radiotherapy for brain metastases. The images show axial T2-WIs (A-D) and coronal CE T1-WIs (E-H); before the WBRT (pre, at age 57) (A, E); at 4 months after the WBRT (B, F); at 7.3 months (C, G); and at 11.4 months (D, H). (A-H) These images are shown at the same magnification and coordinates under co-registration and fusions based on the images before the WBRT. In particular, when comparing the images before and 11.4 months after the irradiation, ventricular dilatations, slightly high-intensity change in the periventricular deep white matters, widening of the cortical sulci, and the Sylvian fissures, suggesting the progression of parenchymal atrophy and degeneration, are observed, which are difficult to explain by tumor shrinkage alone. WI: weighted images; CE: contrast-enhanced; WBRT: whole-brain radiotherapy

Changes in brain morphology and white matter intensities over time from 4 months to 11.4 months after the BM diagnosis are shown in Figure 9.

A decrease in the brain parenchyma volume was evident, which was difficult to explain only by the remarkable shrinkage of multiple BMs, although leukoencephalopathy was inconspicuous [15]. The patient's neurocognitive function continuously improved from before the EBRT for the BMs, at least until her general condition worsened.

## Discussion

Many facilities with a standard linac may only perform WBRT for BMs, such as this case, and observe the responses for 1-2 months [6,15]. Some facilities may adopt dose escalations to 35-40 Gy; however, this inevitably indicates the increased risk of detrimental effects. Tumor responses after WBRT are generally gradual and frequently unfavorable during WBRT, as in this case. Tumor (cyst) expansion and/or aggravation of the surrounding edema frequently occur due to the progress of tumor degeneration and AREs. Resuming and continuing systemic therapy may be abandoned if symptoms do not improve within one month after WBRT completion. Some facilities position SRS as a salvage option for progression after WBRT. Some facilities wait 1-2 months until some degree of tumor shrinkage is confirmed when adding SRS boost following 30-Gy WBRT [6]. Many facilities perform SRS boost in a single fraction with a diverse dose (e.g., 70% dose of 18-25 Gy), causing variable cumulative BED_10_s [16,17]. However, the risk of brain radionecrosis is quite high for the BMs as in this case even with ≥14-15 Gy, while ≤12-13 Gy is insufficient for ensuring excellent tumor control (cumulative BED_10_ ≤65-69 Gy) [7,18]. Meanwhile, some experienced facilities with Leksell Gamma Knife^®^ Icon™ (Elekta, Stockholm, Sweden) which are assertive in treating >10 BMs may attempt fractionated SRS alone. However, the longer the years have elapsed from the cobalt-60 source installation, the longer the treatment time becomes a substantial burden. The efficacy and safety of fractionated SRS alone for multiple BMs totaling >100 cm^3^ remains unclear [7].

WBRT combined with an immediate 5-fr SRS boost with a total BED_10_ of just over 80 Gy was adopted for the BMs in the armamentarium with a conventional linac and CK being available. This scheme resulted in approximately 90% overall tumor reduction and significant improvement in neurocognitive symptoms without brain edema aggravation over 1-6 months after the EBRT completion. The solid components almost disappeared in most of the cystic lesions, although the cystic structures remained, and the enhancements of the cyst walls were markedly reduced. Therefore, the actual tumor reduction rate may be >95%. Appropriate dose fractionation should be adopted in the SRS boost instead of sticking to a single fraction to ensure safety during and after irradiating a total BED_10_ of ≥80 Gy [8-10,19]. In the SRS boost, ≥5 fr appeared appropriate, considering the volume effects from total and individual GTVs and the dose interferences based on the proximity to each other. Further, the resumed chemotherapy after the EBRT may have improved the anti-BM efficacy, and bevacizumab may have attenuated the AREs [4,5,15].

In irradiating multiple lesions simultaneously, treatment planning should be optimized while comprehensively considering the dose interferences to conformally cover the target boundaries, minimize the variation in marginal doses to each target, and efficiently reduce the surrounding brain dose. In this case, the SRS boost using CK was comprehensively optimized based on a single plan utilizing the latest algorithm and the variable aperture collimator. This en bloc consecutive irradiation is extremely efficient as multiple lesions can be irradiated using a single series of robot movements (path) [8,9]. This method is the most suitable and preferred delivery technique for irradiating multiple intracranial lesions using CK, and its widespread application can broaden the indications of CK for approximately 10 BMs.

Another important caveat from this case is that multiple BMs developed without major visceral organ metastases, such as the lungs and the liver, in HR+/HER2- MBC. The BMs can gradually increase during systemic therapy like a silent killer without surrounding edemas and related symptoms, while the growth potentials are somewhat suppressed. Further, the symptoms may become apparent only when the total tumor volume exceeds 100 cm^3^ if the BMs are not localized in the eloquent areas [1-3,5]. Therefore, the head should preferably be scanned at least once every six months when performing a contrast-enhanced computed tomography (CT) scan from the thorax to the pelvis to treat BMs via SRS alone while they are smaller [5]. BMs that require local treatment and are difficult to visualize on CT images can be detected just by acquiring T2-weighted images [1-3,5].

The major limitations of this report include the unknown pathology of all the visible intracranial lesions in the final images and a less than one-year period of image evaluation after the EBRT. These limitations hinder the ability to conclude whether or not the cumulative BED_10_ of 80 Gy is the optimal dose. Lower doses may be sufficient to achieve complete local tumor eradication when combined with sequential chemotherapy, while higher doses may be required to maintain complete responses for >1.5-2 years. Late toxicity, such as brain radionecrosis and white matter damage, generally becomes more apparent after one year [7,8].

In addition, some disadvantages of this treatment scheme include its requirement of 2-3 months to achieve sufficient tumor shrinkage and symptom improvement, and the addition of SRS boost after 30-Gy WBRT substantially increases the surrounding brain doses, despite SRS with extremely steep dose gradients outside GTVs. Tumor regression may be difficult to achieve within one month after the BM diagnosis, and there remains considerable concern regarding late toxicity. Hence, the brain parenchymal volume reduction was evident in less than one year, although neurotoxicity due to chemotherapy may have affected it to some extent [15]. Therefore, simultaneous integration of SRS and WBRT using volumetric-modulated arcs (VMA) may be considered to further increase the efficacy and decrease the surrounding brain doses [14,20]. The WBRT dose in this case can be reduced to approximately 25 Gy (BED_10_ 31.3 Gy; BED_2_ 56.3 Gy) when adding SRS boost to all the 12 lesions, both of which are achieved using VMA [14,15,20]. From 2021 onward, we have primarily adopted the VMA-based ≥10-fr SRS, including non-coplanar arcs with simultaneously integrated reduced-dose WBRT, to more efficiently irradiate and enhance the efficacy and safety for BMs, as in this case.

## Conclusions

WBRT, followed by an immediate 5-fr SRS boost with a total BED_10_ of just ≥80 Gy covering GTVs of large and/or culprit lesions, can be an efficacious and safe treatment option for >10 BMs totaling >120 cm^3^, including multiple large lesions (>10-30 cm^3^) in close proximity. En bloc consecutive irradiation based on a comprehensively optimized single plan is a suitable and preferred delivery technique for treating multiple lesions using CK in terms of irradiation time and comprehensive reduction of normal brain dose. VMA-based SRS with simultaneously integrated reduced-dose WBRT may be an alternative to further enhance efficacy and safety.
